# CT-guided Percutaneous Drainage of Intra-spinal Haematoma as an Alternative to Surgical Evacuation

**DOI:** 10.5704/MOJ.2103.024

**Published:** 2021-03

**Authors:** Peh WCG

**Affiliations:** Department of Diagnostic Radiology, Khoo Teck Puat Hospital, Singapore

Dear Sir,

I read with interest Chung et al’s report of two patients who developed delayed post-operative spinal epidural haematoma after posterior spinal surgery. In both cases, magnetic resonance imaging (MRI) was utilised to accurately diagnose the haematoma compressing the spinal cord. They correctly emphasised the importance of having a high index of clinical suspicion and reported successful outcomes following surgical evacuation of haematoma^1^.

An alternative treatment to consider is imaging-guided percutaneous drainage of the intra-spinal haematoma. This can be achieved using an 18G Tuohy needle, ideally under computed tomography (CT) guidance^2^, although fluoroscopic guidance can also be utilised^3^. Besides epidural hematomas, CT-guided aspiration has also been advocated for decompression of epidural abscesses^4^.

The Tuohy needle is a widely-available standard needle used for epidural access by anaesthetists. This needle has a blunt rounded Huber tip that minimises the risk of damage to vital structures within the spinal canal. The curved tip also allows insertion of a plastic catheter that can be directed, hence enabling a more thorough aspiration of the intra-spinal haematoma beyond the point of the needle puncture site^2^.

Although open surgical drainage is the standard treatment of choice, CT-guided percutaneous drainage of acute or subacute intra-spinal haematoma may be an option where the patient is elderly and/or has high-risk comorbidities whereupon further open surgery and administrating general anaesthesia are not desirable. In the hands of a skilled proceduralist (e.g. musculoskeletal interventional radiologist), this minimally-invasive procedure can be quickly and accurately performed with low risk of trauma to the surrounding structures.
